# The Effect of Lexicality, Frequency, and Markedness on Mandarin Tonal Categorization

**DOI:** 10.3389/fpsyg.2022.836865

**Published:** 2022-07-22

**Authors:** Tzu-Hsuan Yang, Shao-Jie Jin, Yu-An Lu

**Affiliations:** ^1^Department of Linguistics, University of Kansas, Lawrence, KS, United States; ^2^Department of Foreign Languages and Literatures, National Yang Ming Chiao Tung University, Hsinchu, Taiwan

**Keywords:** tonal categorization, tonotactic accidental gaps, lexicality effect, frequency effect, markedness, tonal continua

## Abstract

While the Ganong lexicality effect has been observed for phonemic and tonal categorization, the effects of frequency and markedness are less clear, especially in terms of tonal categorization. In this study, we use Mandarin Chinese to investigate the effects of lexicality, tone frequency and markedness. We examined Mandarin speakers’ tonal categorization of tokens on all possible tonal continua with one end being a word and the other being a tonotactic gap (i.e., an unattested syllable-tone combination). The results of a forced-choice identification experiment showed a general bias against the gap endpoints, with the noted exception of continua involving T4 (X^51^), the most frequent lexical tone. Specifically, when T4 served as the gap endpoint, no obvious bias against it was observed regardless of its lexical status. Moreover, on the T3–T4 continua, there was an apparent bias against T3 (X^214^), the tone with the most complex contour, again, regardless of lexicality, suggesting a strong markedness effect. Taken together, the results of this study show the individual effects of lexicality, tone frequency and markedness, as well as their interactions, which contribute to our understanding of tonal categorization in relation to lexical statistics (tone frequency) and phonology (markedness).

## Introduction

Previous studies have demonstrated that phonetic categorization of an ambiguous sound may be guided by a speaker’s linguistic knowledge. The types of knowledge include, but are not limited to, lexicality, phonotactics, and frequency (e.g., Ganong, 1980; Massaro and Cohen, 1983; Connine et al., 1993; Dupoux et al., 1999; Ahn, 2008; Soo and Babel, 2020). For example, Ganong (1980) employed continua of stops varying in VOT in which one end was a word and the other was a non-word (e.g., *task*-*dask, *tash-*dash*), and asked participants to identify the word they heard. The results showed that English speakers were more likely to identify ambiguous stimuli as the real words (*task* or *dash*) along the continua.

A similar lexical effect has also been observed at the suprasegmental level (Fox, 1984; Fox and Unkefer, 1985; Yang et al., 2019; Soo and Babel, 2020). One such example comes from Mandarin, a tone language with four phonemic tones (high-level Tone 1 [X^55^], rising Tone 2 [X^35^], dipping Tone 3 [X^214^], and falling Tone 4 [X^51^]) that do not necessarily combine with every allowable syllable. For instance, the syllable [ts*^h^*u] can carry T1 ([ts*^h^*u]^55^ “coarse”), T2 ([ts*^h^*u]^35^ “die”) and T4 ([ts*^h^*u]^51^ “vinegar”) but not T3 (*[ts*^h^*u]^214^). Fox and Unkefer (1985) used these syllable-tone combinations that could but do not exist in Mandarin (or *tonotactic accidental gaps*) to examine if the Ganong lexical effect could be observed at the tonal level. They found that when Mandarin speakers were asked to identify T1–T2 continua with word or gap endpoints (e.g., [hei]^55^-*[hei]^35^ “black”-gap; *[s̨ei]^55^-[s̨ei]^35^ gap-“who”), their responses were indeed biased against the gaps. Yang et al. (2019) replicated this experiment with T1–T4 and T1–T2 continua and reported similar results.

Lexical token frequency has also been observed to bias categorization. For example, Connine et al. (1993), using voicing continua ranging from high-frequency words on one end to low-frequency words on the other (e.g., *best*-*pest*), showed that English speakers’ identification was biased toward the high-frequency word endpoints (*best*) (cf. Politzer-Ahles et al., 2020). Word acceptability judgments can also be affected by markedness (Zuraw, 2000, 2002; Frisch et al., 2004; Jin and Lu, 2019). For example, Myers (2015) showed that Mandarin syllables with more marked onsets were more likely to be accepted as words than those with less marked onsets by Mandarin speakers. However, the effects of frequency and markedness on perceptual categorization is less clear, especially at the suprasegmental level.

In this study, we use Mandarin Chinese to investigate the effects of lexicality, tone frequency and markedness. With four lexical tones, there are around 1,500 possible tone-syllable combinations in Mandarin (Lin, 2007); the unattested combinations provide the means to test for lexicality effects involving tone. Note that *frequency* here refers to *tone frequency*, not lexical token frequency, based on the well-established fact that tone can be processed independently from segments (Cutler and Chen, 1997; Lee, 2007; Wiener and Turnbull, 2016; Wiener and Liu, 2021). For a discussion of token frequency effects in Mandarin, see Politzer-Ahles et al. (2020). Previous studies on tonotactic gaps have shown that falling T4 gaps are more readily accepted as words than other gaps (Lai, 2003; Jin and Lu, 2019), while dipping T3 gaps are rated as the least wordlike among all the tones (Jin and Lu, 2019). The general acceptance of T4 gaps has been attributed to T4’s overall higher tone frequency (independent of lexical token frequency), and the fact that the fewest gaps are observed for T4. Based on the *Taiwan Mandarin Conversational Corpus* (Tseng, 2019), T4 has the highest tone frequency (228,182) followed by T3 (129,505), T1 (105,168), and T2 (96,584). On the other hand, T3 has the most marked tonal contour—a complex, dynamic falling-rising tone—compared to the simple contours of rising T2, falling T4, and high level T1. The general rejection of T3 gaps has been attributed to this marked tonal contour (Zhang, 2001).

As the roles of vowels, consonants and tones in understanding and repairing non-lexical items are different (e.g., Wiener and Turnbull, 2016), we cannot assume factors such as lexicality, frequency, and markedness would have the same effects on these unattested syllable-tone combinations. This study examines these factors to understand their effects on tonal categorization.

## Methods

To examine Mandarin speakers’ tonal categorization, we conducted a two-alternative forced-choice identification experiment in which participants were presented with stimuli sampled from 10-step continua for all lexical tone pairs. All data are available in the OSF repository at https://osf.io/ct48a/.

### Participants

Twenty-two Taiwan Mandarin speakers (13 female, 9 male; aged 20–28, *M* = 21.9) were recruited from National Yang Ming Chiao Tung University. The study was conducted in accordance with ethical guidelines approved by the Research Ethics Committee for Human Subject Protection, National Yang Ming Chiao Tung University. None of the participants reported any auditory or visual disabilities. All participants were compensated monetarily for their time.

### Materials

Twelve pairs of Mandarin CV syllables were selected so that each pair contained a subset of the four lexical tones (T1–T2, T1–T3, T1–T4, T2–T3, T2–T4, T3–T4) with a word on one end and a tonotactic gap on the other. Table 1 lists the pairs of stimuli along with the token frequency of the word endpoints.

**TABLE 1:** Stimuli used in the experiment.

T1–T2 continua		T1–T3 continua		T1–T4 continua	
*T1–T2	*[na]^55^-[na]^35^ gap-“take” frequency: 0–225	*T1–T3	*[ny]^55^-[ny]^214^ gap-“girl” frequency: 0–542	*T1–T4	*[ni]^55^-[ni]^51^ gap-“inverse” frequency: 0–15
T1–*T2	[t*^h^*a]^55^-*[t*^h^*a]^35^ “he/she”-gap frequency: 11088–0	T1–*T3	[hγ]^55^-*[hγ]^214^ “drink”-gap frequency: 61–0	T1–*T4	[ha]^55^-*[ha]^51^ “laugh”-gap frequency: 27–0

**T2–T3 continua**		**T2–T4 continua**		**T3–T4 continua**	

*T2–T3	*[k*^h^*u]^35^-[k*^h^*u]^214^ gap-“bitter” frequency: 0–181	*T2–T4	*[t*^h^*γ]^35^-[t*^h^*γ]^51^ gap-“very” frequency: 0–223	*T3–T4	*[hγ]^214^-[hγ]^51^ gap-“congratulate” frequency: 0–5
T2–*T3	[hγ]^35^-*[hγ]^214^ “river”-gap frequency: 464–0	T2–*T4	[tγ]^35^-*[tγ]^51^ “gain”-gap frequency: 844–0	T3–*T4	[k*^h^*a]^214^-*[k*^h^*a]^51^ “card”-gap frequency: 72–0

*T = tonotactic accidental gap.

**FIGURE 1:**
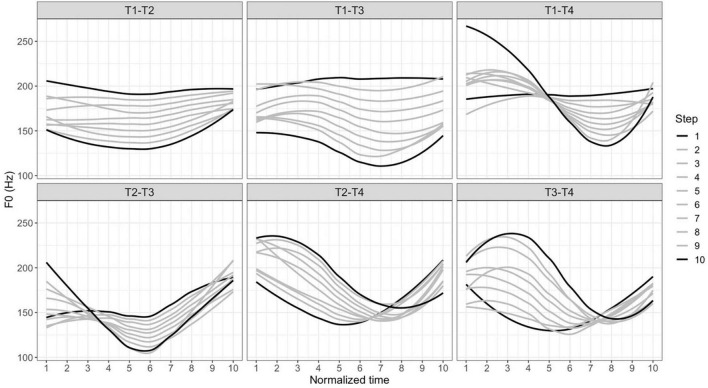
Time-normalized smoothed *f0* trajectories of the resynthesized stimuli (upper panel: T1–T2, T1–T3, T1–T4; lower panel: T2–T3, T2–T4, T3–T4) from steps 1 to 10.

These syllables were naturally produced and recorded by two native Taiwan Mandarin speakers (1 female, 1 male). One token for each syllable was selected from multiple repetitions by three native Taiwan Mandarin speakers to be a good representative of a certain lexical tone. The mean duration of these selected tokens was 512 ms (*SD* = 110 ms). All tokens were scaled to 75 dB using Praat (Boersma and Weenink, 2017). These syllables were then resynthesized using Tandem-Straight, a speech analysis, modification, and resynthesis framework that allows the pitch, duration and voice quality of the entire syllable to be manipulated proportionally (Kawahara et al., 2008), as secondary cues such as duration and creakiness have also been shown to affect listeners’ perception and categorization of tones (Yu, 2010; Wu and Kenstowicz, 2015; Lu and Lee-Kim, 2021). In their work on tonal categorization, Yang et al. (2019) describe the advantages of resynthesizing pitch along with other secondary cues over only resynthesizing pitch. Figure 1 shows the time-normalized *f0* trajectories of the resynthesized stimuli for each tone pair using the Straight algorithm in VoiceSauce (Kawahara et al., 2008; Shue et al., 2011), with the endpoints, Steps 1 and 10, represented with black lines (e.g., on the T1–T2 continua, Step 1 is T1 and Step 10 is T2). The steps in between are represented with gray lines. Previous studies employing a similar task used continua with the number of steps ranging from 6 to 11 (Ganong, 1980; Massaro and Cohen, 1983; Fox and Unkefer, 1985; Dupoux et al., 1999; Ahn, 2008). The three authors listened to all the resynthesized stimuli and decided on the 10-step continua since the difference between each step-wise comparison was sufficiently subtle. Note that these contours are pooled trajectories from the naturally produced tonal endpoints described in Table 1 by two native speakers. Since these endpoints involved different segmental information, we did not impose the same tonal contour onto the same tone.

Note that there are two T3 variants, full T3 (X^214^) and half T3 (X^21^), with the latter variant observed more frequently in Taiwan Mandarin (e.g., Kubler, 1985; Fon and Chiang, 1999). However, the variant with the dipping contour has nevertheless been shown to be the canonical representation of T3 for Taiwan Mandarin speakers (Lu and Lee-Kim, 2021). We thus used the full T3 variant as the stimuli for continuum resynthesis involving T3 (Figure 1, lower panel, leftmost, and rightmost plots) except for those in the T1–T3 continua. The half T3 variant without a final rise was used for the resynthesis of the T1–T3 continua (Figure 1, upper panel, middle plot) since the resynthesis between high-level T1 and dipping full T3 yielded a T2 percept for the ambiguous tokens.

### Procedure

The 240 resynthesized stimuli [6 tonal pairs × 2 talkers × 2 continua (word-gap, gap-word) × 10 steps] were presented in six blocks, with one tonal pair per block, using E-Prime (Schneider et al., 2012). The six blocks as well as the trials in them were randomized. Participants were verbally instructed and given written instructions on the monitor to listen to each stimulus and judge whether they heard the tone displayed on the left (e.g., T1 on the T1–T2 continua) or on the right (e.g., T2 on the T1–T2 continua) by pressing the corresponding key on the keyboard. Participants’ responses were recorded in E-prime. Each block involved a four-trial practice to familiarize the participants with the positions of the labels and task. These practice trials contained only the endpoint stimuli from the target continua. The total duration of the procedure was around 25 min.

### Data Preparation and Analysis

To assess statistical significance, mixed-effects logistic regression modeling was run in *R* (R Core Team, 2017) using the *lme4* package (Bates et al., 2015), and associated *p*-values were obtained using the *lmerTest* package (Kuznetsova et al., 2016). The dependent variable was the Mandarin speakers’ tonal Responses (right-label responses = 1, left-label responses = 0; e.g., T2 on the T1--T2 continua coded as 1 and T1 on the T1--T2 continua coded as 0), and the independent variables were Step (continuous scale, centered), Continua (word-gap vs. gap-word; word-gap as the baseline), Pair (T1--T2, T1--T3, T1--T4, T2--T3, T2--T4, and T3--T4; T2--T4 as the baseline), and the interaction between Continua and Pair.^1^ As the absolute perceptual boundaries along the 10-step continua may differ by tone pair, the relative perceptual boundaries along Step was less relevant (cf. Xu et al., 2006). We thus did not include the three-way interactions among Step-Continua-Pair. Instead, we focused on the *difference* between word-gap and gap-word continua in each tone pair as an indicator for perceptual biases, as demonstrated by the Continua-Pair interaction. The model also included random intercepts for Participant as well as by-participant random slopes for Step, Continua, and Pair.

We can make the following predictions. First, we predict an effect of Step with a positive coefficient—the higher the Step, the more right-label responses should be given by the participants. This would indicate that the participants are doing the task correctly. Second, if the Mandarin speakers’ tonal categorization is mainly guided by lexicality, we would expect to see a general bias against the gap endpoints in their responses. This would be reflected in an effect of Continua with word-gap set as the baseline—we should observe more right-label responses on the gap-word continua than on the word-gap continua. However, if tonal categorization is not only affected by lexicality but also interacts with tone frequency, we would expect to see a weaker effect of Continua for the T1–T4 and T2–T4 pairs in which the most frequent tone is on the right. Specifically, with T2–T4 as the reference, we predict little to no effect of Continua on the T1–T4 pair, and a similar pattern with the T2–T4 pair, as reflected in the lack of a Continua-Pair interaction. The other tone pairs, however, should display a Continua effect due to lexicality (our second prediction), resulting in Continua-Pair interactions between the reference and T1–T2, T1–T3, and T2–T3 pairs. Third, if markedness has an effect on Mandarin speakers’ tonal categorization, we would expect to observe a bias against T3. Specifically, we should see an even larger effect of Continua on T2–T3 and T1–T3 pairs since T3 gap endpoints would be more disfavored, yielding even fewer right-label responses. Finally, the T3–T4 pair will allow us to see the interactions between lexicality, frequency, and markedness. The Continua variable will shed light on the relative effects of lexicality (more right label responses for gap-word than word-gap), frequency (lack of a Continua effect, similar to the T1–T4 and T2–T4 pairs), markedness (a greater Continua effect, similar to the T1–T3 and T2–T3 pairs), and both frequency and markedness (more right label responses for word-gap than gap-word).

## Results

**FIGURE 2:**
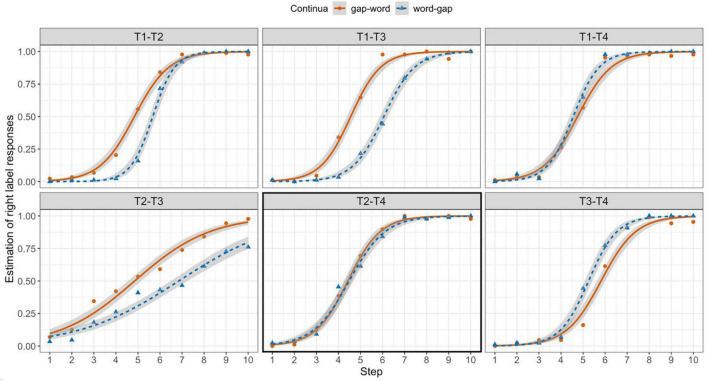
Averaged and estimated right-label responses as a function of Step (1–10) and continua (solid red line for gap-word vs. dotted blue line for word-gap) on different tonal continua (upper panel: T1–T2, T1–T3, T1–T4; lower panel: T2–T3, T2–T4, T3–T4).

The results are shown in Figure 2. The *x*-axis represents the steps on a given tonal continuum. Right-label responses were coded as 1 and the left-label responses were coded as 0. As such, the closer to 1 on the *y*-axis, the more right-label responses were given.

As mentioned earlier, the perceptual boundaries may differ by tone pair. For example, in the case of the T1–T2 pair, a slight change in the level contour of T1 may induce a T2 response and demonstrate an earlier boundary on a T1–T2 continuum. Hence, unlike some previous studies that examine different effects on the *same* tone pair (e.g., Xu et al., 2006), we did not take the midpoint (i.e., Steps 5–6) as an unbiased perceptual boundary. Instead, we considered the *difference* between the gap-word and word-gap continua to be the basis for perceptual bias. We can see that Mandarin speakers’ responses on the gap-word continua (Figure 2, solid lines) involved an earlier shift to the right-label tones in the T1–T2, T1–T3, and T2–T3 panels while those on the word-gap continua (Figure 2, dotted lines) involved a later shift. This pattern demonstrates a general bias against the gap endpoints and can be taken as evidence of the previously reported lexicality effect (Ganong, 1980; Fox and Unkefer, 1985; Yang et al., 2019). This bias, however, was not visually observed for the tonal continua with T4 (T1–T4, T2–T4, and T3–T4). In fact, we observed a *reversed* pattern for T3–T4 continua, with the most marked T3 on the one end and the most frequent T4 on the other. In other words, participants were more likely to categorize ambiguous tokens as T4. The results of the statistical model are summarized in Table 2. In this model, word-gap and T2–T4 (the boxed panel in Figure 2) were treated as the reference levels.

**TABLE 2:** Summary of fixed effects for the model glmer [Response ∼ Step (centered) + Continua (word-gap as reference) * Pair (T2–T4 as reference) + (1 + Step + Continua + Pair | Participant), family = binomial].

	*B*	*SE*	*z*	*p*
(Intercept)	1.23	0.16	1.23	<0.001***
Step	3.42	0.13	3.42	<0.001***
Continua gap-word	0.07	0.18	0.07	0.697
Pair T1–T2	–1.46	0.23	–1.46	<0.001***
Pair T1–T3	–1.87	0.21	–1.87	<0.001***
Pair T1–T4	–0.08	0.19	–0.08	0.692
Pair T2–T3	–2.49	0.38	–2.49	<0.001***
Pair T3–T4	–0.93	0.19	–0.93	<0.001***
Continua gap-word: Pair T1–T2	0.91	0.23	0.91	<0.001***
Continua gap-word: Pair T1–T3	1.69	0.23	1.69	<0.001***
Continua gap-word: Pair T1–T4	–0.30	0.23	–0.30	0.191
Continua gap-word: Pair T2–T3	1.89	0.23	1.89	<0.001***
Continua gap-word: Pair T3–T4	–0.73	0.23	–0.73	0.002**

*p < 0.05, **p < 0.01, ***p < 0.001.

First, we found a significant Step effect with a positive estimate (β = 3.42, *p* < 0.001), suggesting an increase of right-label responses as the stimuli contained more right-label tonal acoustic properties. This indicates that the participants were indeed following the instructions and doing the task correctly. In the T2–T4 panel, no statistical difference between the word-gap (reference level) and the gap-word continua was found (β = 0.07, *p* = 0.697) while significant interactions of Continua*Pair were evident in the other tone pairs, except for T1–T4 (gap-word: T1–T2, β = 0.91, *p* < 0.001; gap-word: T1–T3, β = 1.69, *p* < 0.001; gap-word: T2–T3, β = 1.89, *p* < 0.001; gap-word: T3–T4, β = –0.73, *p* = 0.002). The same asymmetrical pattern (T4 vs. other tones) was also observed for the different tone pairs. We found significant Pair effects for all tone pairs (T1–T2, β = –1.46, *p* < 0.001; T1–T3, β = –1.87, *p* < 0.001; T2–T3, β = –2.49, *p* < 0.001) except T1–T4 (β = –0.08, *p* = 0.692). These robust Continua effects and Continua*Pair interactions for T1–T2, T1–T3, and T2–T3 confirmed the Ganong lexicality effect at the suprasegmental level in which the participants gave significantly more responses that made words and avoided giving responses that made gaps on these continua. The lack of the Continua effect for T2–T4 and the lack of an interaction between gap-word and T1–T4 pair (β = –0.3, *p* = 0.191), on the other hand, were interpreted as participants not being biased against the most frequent T4 despite the fact that the T4 endpoint was a tonotactic gap. The statistical results confirmed our visual observations and our predictions that there was no difference between the gap-word and word-gap continua in the T1–T4 and T2–T4 panels, indicating a tone frequency effect in the realm of tonal categorization. The general preference for T4 seems to be unrelated to the *token* frequencies of the endpoints on these continua. The token frequency of the T4 word endpoint (14) is comparable to that of the T1 word endpoint (27) on the T1–T4 continua, while the token frequency of the T4 word endpoint (233) is much lower than that of the T2 word endpoint (844) on the T2–T4 continua. Nevertheless, no difference was observed between the two tone pairs. The lack of token frequency effects suggests that the leveling of the Continua effect stems from the bias toward the most frequent T4.

Note that, although we did find an interaction between gap-word and T3–T4, the estimate value was negative (β = –0.73, *p* = 0.002), indicating a reversed pattern from the other tone pairs. In the T3–T4 continua, we observed a consistent bias against the marked T3 and a bias toward the frequent T4, regardless of lexicality. This result provides additional support for the frequency effect found in the T1–T4 and T2–T4 panels and further establishes the effect of markedness in tonal categorization. This markedness effect is also supported by the largest estimates in the T1–T3 (β = –1.87, *p* < 0.001) and T2–T3 (β = –2.49, *p* < 0.001) simple effects compared to the other tone pairs.

## Discussion

Taken together, our results can extend the well-established lexicality effect on phonemic categorization to tonal categorization. Moreover, the asymmetrical pattern found on tone pairs involving T4 as a whole, regardless of token frequencies of different tone pairs involving T4, suggests that the robust frequency effect on phonemic categorization was also found at the tonal level in terms of tone frequency. This finding is consistent with previous studies in which T4 gaps were rated as more wordlike than other gaps (Lai, 2003; Jin and Lu, 2019). The general bias against T3 further demonstrated a markedness effect on tonal categorization, an effect that has not been previously reported on tonal categorization. For example, this markedness effect was observed on the T2–T3 continua: when Mandarin listeners were presented with clear T3 tokens (Steps 9–10), they were still reluctant to identify them as T3. This finding, however, needs to be interpreted with caution. On the other two continua involving T3 (T1–T3 and T3–T4), the participants were *not* reluctant to identify clear T3 tokens (Steps 9–10) as T3. The bias was only observed for ambiguous tokens (Steps 4–7). It should be noted that these two tones caused general confusion as indicated by the linear function on the tonal identification of the items on the T2–T3 continua. T3 undergoes a sandhi processes whereby a T3 becomes a T2 before another T3 (T3➔T2/_T3). Previous studies have argued that the sandhi process is one of simplification in that T2, a rising tone, is an articulatorily simpler tone than T3, a dipping tone; otherwise, the two tones are acoustically similar in terms of their rise (Huang, 2001). According to calculations of perceptual distance between tones using multidimensional scaling based on Mandarin speakers’ tonal discrimination response times (Huang, 2001; Hume and Johnson, 2003), the distance between T2 and T3 is the smallest (ΔT2–T3 = 1.596) compared with the other tone pairs (ΔT1–T2 = 1.938; ΔT1–T3 = 1.887; ΔT1–T4 = 1.879; ΔT2–T4 = 1.998; ΔT3–T4 = 1.982). The general confusion presumably arises due to their *acoustic similarity* (Blicher et al., 1990; Shen and Lin, 1991; Shen et al., 1993) and *morphological alternation* between the two tones that has been widely discussed in the literature (e.g., Huang, 2001; Hume and Johnson, 2003). This, however, does not obscure the effect of T3 markedness. Upon hearing acoustically ambiguous T2–T3 tokens, less marked T2 were favored over the more marked T3. Along the T1–T3 and T3–T4 continua, on the contrary, the same bias against T3 was also observed, but only with acoustically ambiguous tokens (Steps 4–7) and not with clear endpoints due to the fact that the endpoint tones were too distinct to be confused.

Strikingly, the effects of markedness and tone frequency may override lexicality to a certain degree. On the T3–T4 continua, regardless of lexicality, the most frequent T4 was favored over the most marked T3. At the segmental level, markedness has been shown to affect speakers’ word acceptance to different degrees based on how wordlike it is (Myers and Tsay, 2005; Myers, 2015). Myers (2015) collected word acceptance judgments from 114 Mandarin speakers who were presented with 3,274 monosyllabic non-words and asked to judge if the stimulus they heard was “like Mandarin” or was “not like Mandarin.” These stimuli were labeled on a continuous scale with their lexical typicality (i.e., how frequent the onsets were in Mandarin) and their onset markedness (i.e., how frequent the onsets were in UPSID, a cross-linguistic phoneme database). An interaction was observed between lexical typicality and markedness in that lexical typicality was stronger with less marked syllables than with more marked syllables. Here, we also found an interaction between markedness and lexicality at the tonal level—regardless of lexicality, the most marked T3 was disfavored in tonal categorization. This markedness effect was also found in other tone pairs, more obviously with the acoustically ambiguous T2–T3 pair and less so with the acoustically distinct T1–T3 and T3–T4 pairs.

Several questions remain. First, one may wonder if the “lexicality effect” displayed in this study could be attributed to token frequency. For example, the difference between T1 and T2 word-gap and gap-word continua may not be due to lexicality but due to the high token frequency of [t*^h^*a]^55^ “he/she” (11,088) on the T1–*T2 continuum vs. [na]^35^ “take” (225) on the *T1–T2 continuum. Given the results from previous studies (Fox and Unkefer, 1985; Yang et al., 2019; Soo and Babel, 2020) and from the other tone pairs involving different endpoint token frequencies in the current study, the lack of lexicality effect is unlikely. That being said, the Ganong type of effect on token frequency (Connine et al., 1993) in tonal categorization requires further research.

One may also wonder if the lack of a lexicality effect in the T2–T4 pair could be attributed to the distinct acoustic differences between the rising and falling tones (ΔT2–T4 = 1.998), and not the preference for the most frequent T4. Although this is a possible explanation given the fact that these two tones involve the largest perceptual distance, we cannot explain the lack of lexicality effect in the other tone pair (T1–T4) that also involves T4, a tone pair that involves shorter perceptual distance (ΔT1–T4 = 1.879).

Observant readers may also have noticed that T3 is the second most frequent tone (section “Introduction”) and asked why, then, T3 would not be favored like T4 was. We propose the stronger effect of T3 markedness overrides the effect of frequency in the cases of tone pairs involving T3. One might also have noticed the T4 responses on clear T4 tokens (Step 9–10) along the *T3–T4 continuum did not quite reach 100% (*M* = 0.95), suggesting a possible unpredicted bias toward T3 and away from T4. We speculate that this was due to creakiness in the natural stimuli (section “Materials”) which biased the participants toward T3 since creakiness is a strong indication for T3 but less so for T4 (Huang, 2019).

Taken together, the results of this study show the individual effects of lexicality, frequency and markedness, as well as their interactions, which contribute to our understanding of tonal categorization in relation to lexical statistics (tone frequency) and phonology (markedness).

## Data Availability Statement

The datasets presented in this study can be found in online repositories. The names of the repository/repositories and accession number(s) can be found in the article/supplementary material.

## Ethics Statement

The studies involving human participants were reviewed and approved by the Research Ethics Committee for Human Subject Protection, National Yang Ming Chiao Tung University. The patients/participants provided their written informed consent to participate in this study.

## Author Contributions

T-HY: conceptualization, methodology, data curation, investigation, writing–review and editing, and visualization. S-JJ: conceptualization, methodology, software, data curation, investigation, and writing–review and editing. Y-AL: conceptualization, methodology, software, validation, investigation, resources, data curation, writing–original draft, writing–review and editing, visualization, and funding acquisition. All authors contributed to the article and approved the submitted version.

## Conflict of Interest

The authors declare that the research was conducted in the absence of any commercial or financial relationships that could be construed as a potential conflict of interest.

## Publisher’s Note

All claims expressed in this article are solely those of the authors and do not necessarily represent those of their affiliated organizations, or those of the publisher, the editors and the reviewers. Any product that may be evaluated in this article, or claim that may be made by its manufacturer, is not guaranteed or endorsed by the publisher.

## Footnotes

1 We made no *a priori* assumptions regarding which tone Pair to set as the baseline. Since the effect of Continua on T1–T4 and T2–T4 (lack of effect) was between that of T1–T2, T1–T3, T2–T3 (positive) and T3–T4 (negative), we chose the one from the first set as the baseline to facilitate the interpretation of the results.

## References

[B1] Ahn M. (2008). “Morphologically conditioned perceptual bias,” in *Proceedings from the Annual Meeting of the Chicago Linguistic Society*, (Chicago, IL: Chicago Linguistic Society), 1–15.

[B2] Bates D. Maechler M. Bolker B. Walker S. (2015). Fitting linear mixed-effects models using lme4. *J. Statist. Softw.* 67 1–48. 10.18637/jss.v067.i01

[B3] Blicher D. L. Diehl R. L. Cohen L. B. (1990). Effects of syllable duration on the perception of the mandarin tone 2/tone 3 distinction: evidence of auditory enhancement. *J. Phon.* 18 37–49. 10.1016/S0095-4470(19)30357-2

[B4] Boersma P. Weenink D. (2017). *Praat: Doing Phonetics by Computer (Version 6.0.26).* Available online at: www.praat.org (accessed October 31, 2017).

[B5] Connine C. M. Titone D. Wang J. (1993). Auditory word recognition: extrinsic and intrinsic effects of word frequency. *J. Exp. Psychol.* 19:81. 10.1037/0278-7393.19.1.81 8423435

[B6] Cutler A. Chen H.-C. (1997). Lexical tone in Cantonese spoken-word processing. *Percept. Psychophys.* 59 165–179. 10.3758/BF03211886 9055613

[B7] Dupoux E. Kakehi K. Hirose Y. Pallier C. Mehler J. (1999). Epenthetic vowels in Japanese: a perceptual illusion? *J. Exp. Psychol.* 25 1568–1578. 10.1037/0096-1523.25.6.1568

[B8] Fon J. Chiang W.-Y. (1999). What does chao have to say about tones? A case study of Taiwan Mandarin. *J. Chin. Linguist.* 27 13–37.

[B9] Fox R. A. (1984). Effect of lexical status on phonetic categorization. *J. Exp. Psychol. Hum. Percept. Perform.* 10 526–540. 10.1037/0096-1523.10.4.526 6235317

[B10] Fox R. A. Unkefer J. (1985). The effect of lexical status on the perception of tone. *J. Chin. Linguist.* 13 69–90.

[B11] Frisch S. A. Pierrehumbert J. B. Broe M. B. (2004). Similarity avoidance and the OCP. *Nat. Lang. Linguist. Theory* 22 179–228. 10.1023/B:NALA.0000005557.78535.3c

[B12] Ganong W. F. (1980). Phonetic categorization in auditory word perception. *J. Exp. Psychol. Hum. Percept. Perform.* 6 110–125. 10.1037/0096-1523.6.1.110 6444985

[B13] Huang T. (2001). “The interplay of perception and phonology in tone 3 sandhi in Chinese Putonghua,” in *Studies on the Interplay of Speech Perception and Phonology*, Vol. 55 eds Hume E. Johnson K. (Columbus, OH: Ohio State University), 23–42.

[B14] Huang Y. (2019). “The role of creaky voice attributes in Mandarin tonal perception,” in *Proceedings of the 19th International Congress of Phonetic Sciences*, eds Calhoun S. Escudero P. Tabain M. Warren P. (Melbourne, Vic: Australasian Speech Science and Technology Association), 1465–1469. 10.1121/10.0000721

[B15] Hume E. Johnson K. (2003). “The impact of partial phonological contrast on speech perception,” in *Proceedings of the 15th International Congress of Phonetic Sciences*, eds Sole M. J. Recasens D. Romero J. (Barcelona: Universitat Autónoma de Barcelona), 2385–2388.

[B16] Jin S.-J. Lu Y.-A. (2019). “The roles of duration, rhyme structure and frequency in mandarin accidental gaps,” in *Proceedings of the 19th International Congress of Phonetic Sciences*, eds Calhoun S. Escudero P. Tabain M. Warren P. (Melbourne, Vic: Australasian Speech Science and Technology Association), 2032–2035.

[B17] Kawahara H. Morise M. Takahashi T. Nisimura R. Irino T. Banno H. (2008). “Tandem-STRAIGHT: a temporally stable power spectral representation for periodic signals and applications to interference-free spectrum, F0, and aperiodicity estimation,” in *Proceedings of the 2008 IEEE International Conference on Acoustics, Speech and Signal Processing*, (Piscataway, NJ: IEEE), 3933–3936. 10.1109/ICASSP.2008.4518514

[B18] Kubler C. C. (1985). The influence of Southern Min on the Mandarin of Taiwan. *Anthropol. Linguist.* 27 156–176.

[B19] Kuznetsova A. Brockhoff P. B. Christensen R. H. B. (2016). *LmerTest: Test in Linear Mixed Effects Model: R Package Version. 2.0–33.*

[B20] Lai Y. C. (2003). *A Perceptual Investigation on Mandarin Tonotactic Gaps.* Hsinchu: National Tsing Hua University.

[B21] Lee C.-Y. (2007). Does horse activate mother? Processing lexical tone in form priming. *Lang. Speech* 50 101–123. 10.1177/00238309070500010501 17518105

[B22] Lin Y.-H. (2007). *The Sounds of Chinese.* Cambridge: Cambridge University Press.

[B23] Lu Y.-A. Lee-Kim S.-I. (2021). The effect of linguistic experience on perceived vowel duration: evidence from Taiwan Mandarin speakers. *J Phon.* 86:101049. 10.1016/j.wocn.2021.101049

[B24] Massaro D. Cohen M. (1983). Phonological constraints in speech perception. *Percept. Psychophys.* 34 338–348. 10.3758/BF03203046 6657435

[B25] Myers J. (2015). Markedness and lexical typicality in Mandarin acceptability judgments. *Lang. Linguist.* 16 791–818. 10.1177/1606822X15602606

[B26] Myers J. Tsay J. (2005). “The processing of phonological acceptability judgments,” in *Proceedings of Symposium on 90-92 NSC Projects*, Taipei, Taiwan, 26–45.

[B27] Politzer-Ahles S. Lee K. K. Shen L. (2020). Ganong effects for frequency may not be robust. *J. Acoust. Soc. Am.* 147 EL37–EL42. 10.1121/10.0000562 32007024

[B28] R Core Team (2017). *R: A Language and Environment for Statistical Computing.* Vienna, Austria. Available online at: http://www.R-project.org/: R Foundation for Statistical Computing (accessed April 11, 2020).

[B29] Schneider W. Eschman A. Zuccolotto A. (2012). *E-Prime User’s Guide.* Pittsburgh, PA: Psychology Software Tools Inc.

[B30] Shen X. S. Lin M. (1991). A perceptual study of Mandarin tones 2 and 3. *Lang. Speech* 34 145–156. 10.1177/002383099103400202

[B31] Shen X. S. Lin M. Yan J. (1993). F 0 turning point as an F 0 cue to tonal contrast: a case study of Mandarin tones 2 and 3. *J. Acoust. Soc. Am.* 93 2241–2243. 10.1121/1.406688

[B32] Shue Y. L. Keating P. Vicenik C. Yu K. (2011). Voicesauce: a program for voice analysis [computer program]. *J. Acoust. Soc. Am.* 126:2221. 10.1121/1.3248865

[B33] Soo R. Babel M. (2020). “Lexical competition affects Cantonese tone mergers in word recognition,” in *Poster Presentation at 17th Conference of the Association for Laboratory Phonology (LabPhon17)*, Vancouver, BC, Canada.

[B34] Tseng S.-C. (2019). “ILAS Chinese Spoken Language Resources,” in *Proceedings of LPSS 2019*, Taipei, 13–20.

[B35] Wiener S. Liu J. (2021). Effects of perceptual abilities and lexical knowledge on the phonetic categorization of second language speech. *JASA Express Lett.* 1:045202. 10.1121/10.0004259 36154204

[B36] Wiener S. Turnbull R. (2016). Constraints of tones, vowels and consonants on lexical selection in Mandarin Chinese. *Lang. Speech* 59 59–82. 10.1177/0023830915578000 27089806

[B37] Wu F. Kenstowicz M. (2015). Duration reflexes of syllable structure in Mandarin. *Lingua* 164 87–99. 10.1016/j.lingua.2015.06.010

[B38] Xu Y. Gandour J. T. Francis A. L. (2006). Effects of language experience and stimulus complexity on the categorical perception of pitch direction. *J. Acoust. Soc. Am.* 120 1063–1074. 10.1121/1.2213572 16938992

[B39] Yang T.-H. Jin S.-J. Lu Y.-A. (2019). “The effect of Mandarin accidental gaps on perceptual categorization,” in *Proceedings of the 19th International Congress of Phonetic Sciences*, eds Calhoun S. Escudero P. Tabain M. Warren P. (Melbourne, Vic: Australasian Speech Science and Technology Association), 2022–2026.

[B40] Yu K. M. (2010). “Laryngealization and features for Chinese tonal recognition,” in *Proceedings of the 11th International Speech Communication Association*, eds Kobayashi T. Hirose K. Nakamura S. (Makuhari: International Speech Communication Association (ISCA)) 1529–1532. 10.21437/Interspeech.2010-446

[B41] Zhang J. (2001). *The Effects of Duration and Sonority on Contour Tone Distribution: Typological Survey and Formal Analysis*. Ph.D. thesis, University of California, Los Angeles, CA.

[B42] Zuraw K. (2000). *Patterned Exceptions in Phonology.* Ph.D. thesis, University of California, Los Angeles, CA.

[B43] Zuraw K. (2002). Aggressive reduplication. *Phonology* 19 395–439. 10.1017/S095267570300441X

